# Professionalisation of International Medical Volunteer Work to Maintain Ethical Standards: A Qualitative Study Exploring the Experience of Volunteer Doctors in Relation to UK Policy

**DOI:** 10.3390/medsci7010009

**Published:** 2019-01-14

**Authors:** Holly Eadsforth

**Affiliations:** Department of Anaesthetics, Glasgow Royal Infirmary, Glasgow G4 0SF, UK; holly.eadsforth@nhs.net; Tel.: +44-141-211-4000

**Keywords:** volunteer, global, international, medical, ethics, policy

## Abstract

Doctors from the United Kingdom are increasingly involved in international medical volunteerism in low- and middle-income countries (LMICs). Although supported by government policy this practice lacks infrastructure and coordination. Volunteer activities can have positive impact but also risk causing harm. Without external governance the responsibility lies with volunteers and their organisations to self-evaluate their activities. This study aimed to explore influences affecting volunteer engagement with ethical standards and evaluative practice. Semi-structured interviews were conducted with seven doctors working in the Scottish National Health Service with volunteer experience in LMICs. Findings were analysed thematically to explore this issue in view of ongoing policy development. Although ethical standards were valued by participants they were unaware of relevant government policy. Influences on volunteer development are unstructured and vary in quality. Evaluation lacks structure and framing. Volunteer physicians face a number of barriers to engaging in critical evaluation of their activities in LMICs. Development and professionalization of medical volunteering in LMICs needs to address volunteer preparation and evaluative practice to maximise the benefits of volunteering, reduce the risk of harm and maximise learning and accountability. Further areas of research are suggested to inform professionalisation of this sector.

## 1. Introduction

The practice of healthcare professionals volunteering in low and middle-income countries (LMICs) has become a recognised part of the United Kingdom’s (UK) global health contribution. It has been described in government policy as having a key role in international healthcare development [1] and predicted to become the ‘norm not the exception’ [2] (p. 5).

There are no formal statistics to quantify the number of physicians from the UK volunteering overseas. However, in a 2016 study of 911 National Health Service (NHS) staff, 42% reported they had experience overseas either volunteering or as a student [3]. The Tropical Health Education Trust reported involvement with over 2000 NHS workers as part of their volunteer partnership programmes [4]. A recent estimate of volunteer activity in LMICs by doctors from the United States suggested that this could represent an economic investment of $3.7 billion [5]. Although on a smaller scale, UK costs are likely to be significant, particularly when compared to the most recent total budget for bilateral overseas development aid for health of £1003 million in 2015 [6]. The scale of volunteer activities cannot only be measured in economic terms; Caldron et al. [7] describe how these activities comprise one part of a country’s wider global political engagement and highlight the fact that medical volunteerism carries diplomatic as well as economic value.

Despite the scale of investment volunteer opportunities can vary widely and the sector lacks infrastructure and coordination. A wide range of volunteer opportunities exist in surgery, dentistry and medicine, from large to small organisations [8]. The Academy of Medical Royal Colleges [9] reported that these opportunities are often fragmented, poorly coordinated and volunteers may be lacking in information. There is an overall lack of standardisation in engaging volunteers, pre-departure training, support and debrief.

While medical volunteerism has potential for positive impact, the risk of harm to patients, institutions and communities from recipient countries is also well recognised [10,11]. Clinical benefit may be less than anticipated due factors such as different diseases or patient demographics and limited follow-up [12]. Patients may even come to direct clinical harm due to acceptance of lower quality standards or volunteers acting beyond their competency. Critics have also described a risk of social harm, for example language barriers or cultural incompetency impacting on the patient physician relationship [13,14] or on a broader scale the perpetuation of structural violence through reinforcement of pre-existing power imbalances [15]. This has resulted in a number of ethical standards being proposed in academic literature [16,17,18] and by the UK government [1]. Although developed within different contexts, broad themes are similar: partnership, sustainability, education, preparation and evaluation of impact.

Anecdotal evidence of unethical and harmful practice suggests there is potential for a gap to open between these proposed ethical standards and the reality of volunteer practice on the ground. To prevent this there is a need for volunteers to develop an awareness and understanding of the ethical standards which are required. Developing a personal ethical framework is a complex process and likely to be influenced by multiple factors including previous educational and clinical experiences. Little research has been done on these influences and processes of how physicians develop their understanding of core ethical standards for volunteer work in LMICs.

Volunteer physicians must also be prepared to undertake critical self-appraisal to maintain the ethical quality of their activities. Government policy calls for active engagement in critical reflection and evaluation for learning and accountability [2]. Médecins Sans Frontières (MSF) agree that evaluation is necessary for transparency and accountability [19]. They maintain that it is a key mechanism to keep global health interventions on track both operationally and in terms of organisational values. Reflective and evaluative practice may also contribute to a volunteer physician’s ethical framework as they learn from experience. Systematic reviews of literature regarding short-term medical volunteerism have found that rigorous evaluation is scarcely published [7,20,21,22]. Frameworks have been developed to guide reflection [23] and evaluation [16], however there is little evidence that these are being actively used. There has been little research exploring potential barriers and opportunities to participating in critical evaluation.

The Scottish government is planning to develop international medical volunteering in the wake of their refreshed International Development Strategy [24] which addresses Scotland’s contribution towards the Sustainable Development Goals. They requested a report exploring the current state of volunteering, which was recently published by the Royal College of Physicians and Surgeons of Glasgow, entitled ‘Global Citizenship in the Scottish Health Service’ [25]. This report highlighted the need to develop policy and infrastructure to professionalise medical volunteerism in LMICs to maximise benefits to partners in LMICs as well as the NHS.

The current lack of infrastructure and standardised pathways for UK medical volunteers is no longer universal to all work in LMICs. The development of the UK International Emergency Trauma and Medical Registers (UKIETR) have made significant steps towards coordination and professionalisation of UK physicians in humanitarian disaster response [26]. This body has created a register for UK medical volunteers under one organisation to improve the quality, coordination and governance of the UK response. Through this they are able to deliver a more organised approach to training and pre-departure simulation as well as focusing on team based competencies to improve their performance [27]. They also recognise the value of evaluation and accreditation, supervision of less experienced volunteers and post-trip debriefing as part of their professionalisation. Furthermore, Wall [15] and DeCamp [11] highlight a stark contrast between the rigor of governance in international medical volunteering compared to medical research in LMICs. In addition to being strongly advocated in UK government policy [28], familiarity with ethical standards in a research setting is a legal requirement [29]. Individuals can access appropriate training in-person or online to learn about Good Clinical Practice standards. There is no equivalent training requirement or governance framework for international medical volunteering. This lack of professionalisation is also more widely relevant to the rest of the UK and other countries supplying medical volunteers to LMICs. Lasker [30] undertook research of 177 organisations involved in medical volunteering in the US. She found that volunteer organisations often have competing interests and incentives which may obscure and detract from their focus on optimally designing projects to meet the needs of LMICs. This further highlights the need for individual physicians to be able to make wise judgement when investing their time and expertise in a field which has been described as intrinsically ethical not neutral in nature [10].

A literature review was undertaken, using the Ovid MEDLINE® ALL database (1946 to 10 March 2017) (https://ovidsp.ovid.com/). The literature search used combinations of the following terms (with synonyms and closely related words): “medical,” “mission,” “trip,” “brigade,” “foreign” “overseas,” “international,” “volunteer,” “short-term,” “ethics.” Further publications were identified by examining the reference lists of all included articles and searching relevant websites and grey literature.

**Figure 1:**
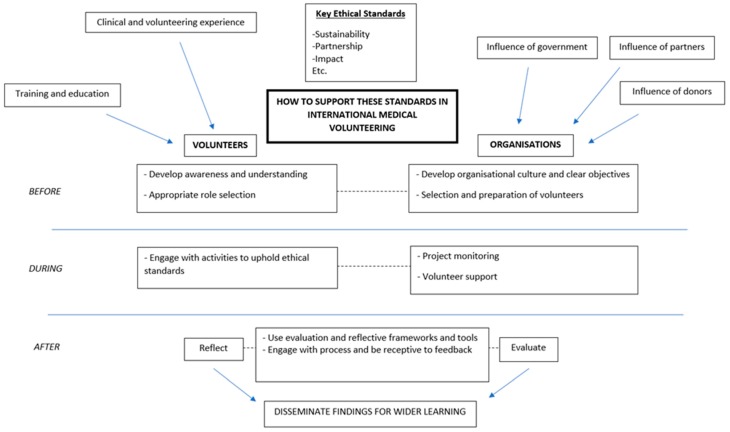
Processes influencing the maintenance of ethical standards in volunteering in low- and middle-income countries (LMICs).

This literature review established that key ethical quality standards for international medical volunteerism have been discussed in academic literature and at a policy level, although no universal guidelines currently exist. Furthermore, the application of these ethical standards in practice is not well documented. While self-evaluation of volunteer activities is an essential step to maintain ethical standards, it is rarely documented and may not be taking place. In the absence of external regulation, the process of upholding ethical standards requires effective engagement and collaboration between volunteers and their organisations. The framework in Figure 1 was developed by the author from the literature review to represent the range of processes involved.

This research aims to investigate some of the knowledge gaps around this issue. Qualitative interviews were conducted with volunteer doctors from the Scottish NHS to explore volunteer development and engagement with ethical standards as well as their experience of debrief and evaluation processes.

## 2. Materials and Methods

A qualitative approach was chosen to explore the perceptions and lived experience of volunteer physicians. Two main aims were identified to explore this theme of ethics of international medical volunteerism and self-evaluation from a volunteer perspective:

1. Volunteer development and engagement with ethical standards.
2. Issues influencing the evaluation of their activities

Recruitment was through a mixture of purposive and snowball sampling. Participants were required to have had experience as a fully qualified clinician of volunteering in LMICs. Volunteers who had experience of only military, expedition or acute disaster relief were excluded as these represented different contexts to those explored in the literature review. To mitigate against recall bias, only participants with experience within the last five years were included.

Semi-structured interviews lasting 30–50 min were conducted between April and May 2017. One interview was via telephone, the rest were face-to-face. They were audio recorded and transcribed verbatim. Written consent was obtained from each interviewee prior to the interview taking place. Ethical approval was obtained from the University of Manchester (Ref: 2017-1696-2722) as well as the NHS Research and Development Board (IRAS project: 224653).

The framework in Figure 1 was used to structure an interview guide (see Appendix A) and address the main themes although not all aspects could be addressed in the scope of this research. Questions were adapted to each interviewee. Participants were asked to provide context to their answers by describing specific examples of their experiences as well as volunteer preparation, educational and clinical experiences which may have influenced them. For analysis, the 4-stage approach outlined by Green et al. [31] of immersion, coding, categorisation and theme identification was used. NVivo was used to facilitate coding of transcripts. Although coding was approached inductively to allow for new and unexpected findings to emerge from the data, the framework from Figure 1 was considered during categorisation. The analysis involved comparing and contrasting data from individuals as well as between different participants to establish conflict and agreement within the transcripts. Thematic analysis used secondary research on social theory to explore and interpret these findings and establish possible implications in the discussion.

## 3. Result

Seven participants were recruited and interviewed from across NHS Scotland. The participants represent a range of volunteer experience and clinical seniority (see Table 1) This reflects the variety of volunteer opportunities available and the diverse backgrounds of volunteer physicians. There were a variety of reasons which motivated these doctors to volunteer in LMICs which ranged from clearly defined roles to a more general personal interest in volunteering. Participants #5 and #7 had been invited by colleagues to volunteer in specific roles based on their skillset, which included teaching or service development. Participant #3 applied for his position as a training opportunity provided by his specialist postgraduate college. The remaining volunteers described motivations including the desire to experience different cultures (#1), gain more clinical experience (#2) and to have impact and ‘make a difference’ (#4,6).

### 3.1. Volunteer Engagement with Ethical Standards

The literature review provided anecdotal evidence of volunteers who were clearly lacking in awareness and/or engagement with ethical risks and standards of international medical volunteerism. Subsequently, one of the research objectives was to explore whether volunteers were aware of the ethical principles involved in their volunteer activities and whether their understanding and interpretation of these principles reflected current academic and policy discourse. All of these interviewees demonstrated high regard for maintaining core ethical standards, describing them as ‘critical,’ ‘crucial,’ ‘important’ and ‘highly relevant.’ Their interpretation of these standards was in keeping with recognised definitions outlined in academic literature. For example, sustainability requiring mutual engagement and ownership:

“I suppose in terms of sustainability it’s about getting people to buy into it and take ownership of it.”

#3

All of the participants also showed that they recognised the potential for lack of benefit or harm resulting from poorly designed or executed volunteer activities.

“If you look then [volunteer physicians] have been doing this for decades now and actually the situation’s still pretty bad.”

#6

“We’re not leaving half of [these donations] because it’s not going to help, its actually just going to make things worse.”

#1

“[…] you see the damage that does to places by people wanting to do good but not the end outcomes not being anything near as beneficial as people anticipate.”

#5

However, none of the participants were familiar with any of the frameworks for ethical engagement found in the literature review, including ethical standards for volunteer engagement published by the government [1].

### 3.2. Influences on Volunteer Development

A variety of possible influences on volunteer development of an ethical framework were discussed by participants. These included formal undergraduate and postgraduate training, preparation from volunteer organisations and informal influences.

None of the participants felt that their undergraduate educational experience had prepared them well for volunteering in LMICs. As postgraduates, two interviewees had done the Diploma in Tropical Medicine & Hygiene which they described as a valuable experience and Participant #3 had also taken a short postgraduate course on his specialty in LMICs. This individual had also received more extensive training prior to his volunteer placement which had been co-ordinated by his specialist college. He described this as a ‘solid’ preparation for his voluntary experience. Participant #7 felt that the ‘Good Clinical Practice ’clinical research training mentioned in the literature review had helped provide him with an ethical framework which he could then apply to volunteering.

Training received from volunteer organisations varied widely. Two participants noted time constraints which they felt limited the preparation they received. Participant #1 received on the job training from other volunteers who had only been in-country for a week or two and was quickly expected to provide this herself for subsequent volunteers. Participant #6 described his preparation as ‘pretty shocking.’ They also described that their training focused exclusively on logistics rather than ethical standards or overall objectives. These experiences contrast to Participant #2 who had lengthy telephone discussions with one of the organisation’s trustees who used this opportunity to share their strategic vision of the organisation and ethical standards with him.

Five of the participants mentioned more experienced colleagues who had a significant impact on their development as a volunteer. This had taken various forms either through sharing attitudes and values, providing networking opportunities or by guiding them through cultural obstacles. Participants described these influential characters positively, as providing ‘eye-opening’ or ‘valuable’ insights or experiences, having a ‘nurturing’ role and often they had long-term contact with their role models, either socially or in a work context.

A variety of informal social networks were described which had allowed interviewees to meet colleagues who had similar experiences. These included university global health departments, specialist colleges, friendship groups established from previous volunteer experience and professional courses. One interviewee even discussed how the technical department in his hospital represented a common point of contact for doctors who volunteered abroad as they would encounter each other while salvaging equipment for their missions.

Interviewees described different effects from these peer interactions. Some interviewees described ‘bouncing ideas’ off colleagues during their development of projects in LMICs. Some requested specific advice about a peer’s experience in a certain role or organisation. Two participants demonstrated active and critical reflection on their colleagues’ experiences which influenced their understanding of what makes a volunteer project ethical and effective:

“[I discuss] with people and think ‘Oh well, you know, what did I like the sound of, what did I not like the sound of.’ And then you know I suppose from there, you then form realisation of well, you know, what is it about the things that sound good that make them sound good and what is it about the things that sound like a disaster that make them sound like a disaster.”

#2

### 3.3. Debrief and Evaluation

#### 3.3.1. Structure and Framing

Interviewees had encountered a wide range of evaluation processes. Some had regular meetings throughout their volunteer placement, where others gave feedback at the end. Often evaluation was multi-modal in the form of both written and verbal feedback. Questions provided by organisations for written feedback were described as ‘broad’ and ‘generic’:

“[…] it was just ‘What went well, what didn’t? What could be improved?’ I don’t think there was anything particularly… Yeah it didn’t really ask a great deal of specifics.”

#7

This ‘open’ approach to evaluation was described by five of the participants. In contrast, Participant #3 described how a structured debrief forced him to confront more difficult questions.

“I think the structured stuff was really useful in that it asked questions that I had to answer and I wasn’t able to shy away from, that I was just caught up for some time with.”

#3

Participants were unclear in many examples about the purpose of feedback or reports they had provided to their organisations.

“It wasn’t sort of as ‘This is a debrief’ but I guess that’s what it was.”

#1

The debrief experiences described by volunteers covered a range of issues as well as or instead of evaluating the quality standards of their activities. These included feedback on logistical factors (e.g., accommodation and transport), personal reflection on what they had learned from the opportunity, psychological or emotional support, clinical case reports and gathering evidence to support future funding opportunities.

#### 3.3.2. Barriers to Participation in Critical Evaluation

Volunteers indicated that they valued reflective practice and critical evaluation but that this process was not prioritised compared to other commitments:

“[…] things like that are important but non-urgent, so they never get done.”

#4

Two participants involved at a managerial level with their volunteer activities described ‘moving forwards’ to plan future activities as a high priority which reduced time available for reflection on ongoing or completed projects.

Another barrier frequently described was the perceived difficulty of assessing the impact of their activities:

“Have I done anything sustainable, at all? And sometimes I look at things and just think ‘What did I actually do?’”

#3

“This qualitative stuff is much harder work and not really my skill, at all.”

#4

Interviewees described the challenge in evaluating outcomes that were ‘indirect,’ ‘on a societal level’ and ‘hard to measure.’ This compared this to quantitative evidence which was described by more than one interviewee as more ‘tangible’:

“Yeah so the clinical experience itself was doing good. But obviously it was on a much smaller, a much lower level than anything educational would.”

#2

Participants described difficulty in finding appropriate forums to share their critical evaluation findings to support wider organizational learning:

“I think these experiences are so multifaceted and so. you know it’s very difficult to present it all in a written document.”

#3

“It would be too soft in points […] it’s not really scientific, in inverted commas, what people want.”

#5

## 4. Discussion

This study aimed to address some of the knowledge gaps around how to maintain ethical standards of international medical volunteering from the perspective of volunteer doctors. This is a topical issue gaining increasing traction in political discourse and currently undergoing development in Scotland [25]. The findings from this research highlight a number of areas for consideration in the professionalisation of medical volunteerism in the Scottish NHS and on a wider scale. The broad spread of background and experience even within a small sample demonstrates the varied nature of volunteer work undertaken by physicians of in LMICs.

It is reassuring that the volunteers in this study recognised and valued the need to maintain ethical standards in their practice in LMICs. However, they were unaware of existing policy regarding quality standards of volunteering. This included consultants with many years’ experience of volunteering in LMICs, as well as doctors within a few years of graduation. In the absence of structured training or clear frameworks which effectively communicate policy objectives to a volunteer level, volunteers are developing their own personal ethical frameworks through a number of informal mechanisms including social networks and role-models. These influences lack standardization and are not necessarily aligned with Scotland’s development policy. This may result in physician’s participating in activities which have limited benefit to partners in LMICs or the NHS and may even risk harm. Mano-Negrin and Mittman [32] highlight the importance of informal and unstructured social networks on physician behaviour in the context of clinical decisions. and suggest that these may be a powerful tool for the dissemination of clinical guidelines. The ‘Global Citizenship’ report [25] outlines plans emerging in Scotland to formalise and expand existing global health networks. This could represent an opportunity to disseminate structured ethical frameworks and offer an alternative to traditional educational methods at bridging the gap between policy and practice.

Influential role-models were also described by participants as having a key role in shaping their understanding of the ethical implications of volunteer work in LMICs. Role-modelling represents a well-recognised form of professional development in the medical field, although Paice et al. [33] describe how it is not a dependable way of imparting attitudes and values as some senior clinicians may display poor attitudes and unethical behaviour. The development of mentorship programmes could offer another strategy to facilitate this transfer of knowledge and values from more to less experienced medical volunteers. These potential opportunities for standardization and development of influences on volunteer development through alternative education and dissemination methods warrant further research.

There is little evidence of active critical evaluation of medical volunteer activities in LMICs in academic literature. This study identified that volunteer physicians are engaging in evaluative practice to some extent. However, this process may lack enough structure to focus on meaningful aspects of volunteer activity. For comparison, see Table 2 which compares the debrief experience of these participants to the list of key evaluation questions from MSF’s evaluation handbook [19] which examine specific aspects of their programs in relation to ethical standards. Participants described many potential areas for discussion as part of a ‘debrief’ experience which do not necessarily facilitate critical evaluation of quality standards. Without adequate structure and framing, evaluation processes risk being superficial and failing to address whether quality standards are fundamentally being upheld.

Possible barriers which may impede volunteer engagement in evaluation were also identified. Deprioritization, perceived lack of ability to judge impact and difficulty in communicating findings to others may contribute to the absence of critical evaluation in the literature. Ackers and Ackers-Johnson [34] argue that the current approach to evaluation is ultimately restricted within a medical science paradigm which leads to inappropriate focus on quantitative results and neglect of more complex social issues at stake. This was reflected in the experience of these participants. Efforts to increase evaluation and monitoring as a method to professionalise volunteering may prove ineffective if these barriers to engagement are not addressed.

This research is on a small scale and in the particular context of NHS Scotland, therefore findings should be regarded as preliminary and exploratory in nature. There is still further research to be done to explore this issue which could help further establish the key factors involved in volunteer development and inform developing government policy, programmes and recommendations. The perspectives of LMIC partners, volunteer organisations or other cadres such as volunteer allied health professionals are also clearly relevant and would give a more comprehensive view of this issue.

Overall this research has explored some of the less well understood areas and processes which underpin the ethical standards of international medical volunteering. Volunteer activities are occurring on a large scale with significant political and financial investment. Despite the potential for limited benefit or even harm, there is limited evidence of ethical engagement and evaluation from volunteers and their organisations in the literature. The lack of standardization and coordination of these activities in general contrasts to established governance systems in the UK for research in LMICs and more recent developments in professionalizing the medical humanitarian response. The potential gap between policy and practice, the influence of role models, a lack of structure and framing for debrief as well as barriers to engagement in evaluation are all issues which may be generalisable to the medical volunteer force on a larger scale. These findings highlight the need to develop guidance on best practice in volunteer work in LMICs which is publicized effectively to volunteer physicians. Evaluation of volunteer activities also requires scrutiny to ensure aspects of ethical quality are appropriately addressed. Professionalisation is necessary to maximise benefits and avoid harm for both partners in LMICs and the NHS. Further research is needed to help guide this development to ensure ethical standards are upheld.

## Figures and Tables

**Table 1:** Participant demographics.

Participant	Grade (at Time of Interview)	Type of Volunteering	Volunteer Organisation	Volunteering Experience
1	5 years postgraduate	Direct patient care, education	NGO	Two (2-week and 1 month)
2	3 years postgraduate	Direct patient care, education	NGO	3 months
3	7 years postgraduate	Direct patient care, quality improvement, education	Postgraduate college	6 months
4	Consultant	Innovation, research, education	Institution level partnership with external funding	>10 years on multiple projects
5	Consultant	Service development, education	NGO	Short-term visits with long-term partnership >7 years
6	3 years postgraduate	Direct patient care	NGO	3 months
7	4 years postgraduate	Education	Institution level partnership with external funding	2 weeks

NGO—non-governmental organization.

**Table 2:** Comparison of Médecins Sans Frontières (MSF) Evaluation Framework [19] with participant debrief.

MSF Key Evaluation Questions [19]	Participant Experience of Debrief
1. Relevance “Are we addressing the real needs?”	1. What went well?
2. Appropriateness “Have things been done right?”	2. What didn’t go well?
3. Effectiveness “Have the right things been done?”	3. What could be improved?
4. Efficiency “Were things done in the best possible way?”	
5. Impact “Is the programme making a difference?”	
6. Continuity “Was the assistance provided in a way that took account of the longer-term context?”	

## Appendix A. Interview Guide

### Appendix A.1. Introduction

What motivated you to get involved in volunteering in LMICs in the first place? How did you end up getting involved with the projects you’ve volunteered with?

### Appendix A.2. Understanding of Ethics

Before you started volunteering did you have much idea about what would make a volunteer project more or less worthwhile? Has your opinion on that changed over time?

Are you aware of any standards for good (ethical) practice in STMMs? How do you think you have developed your understanding of these concepts?

Did you have training from the volunteer organisation before your STMM? Were they clear as an organisation about the ethical standards they were aiming to achieve?

Are there any other sources where you developed understanding of ethical concepts? Examples. Medical school? Postgrad training? NHS experience? Learning from previous STMMs? Informal from friends/colleagues? Which of these had the biggest influence? Has your understanding changed over time?

Do you think everyone involved in your last STMM had a shared understanding of these key concepts?

If you were volunteering with a colleague who didn’t seem to recognise any need for ethical standards like sustainability how would you react?

Do you think your awareness or understanding of these was influenced by your last STMM?

### Appendix A.3. Evaluation

Have you had the opportunity to debrief on your volunteer experience with your volunteer organisation? How have you done this? (formal/informal) (written tools?) What was the main purpose of this debrief? If it didn’t happen why not?

Have you had any experiences volunteering where you felt the mission wasn’t meeting ethical standards? How did you handle it? Was this addressed in your debrief?

How was feedback received? (or) Why didn’t you say anything? Have you ever had experience of feeding back on problems during your routine work in the NHS? How did this compare?

Have you had the opportunity for informal reflection on volunteer work when talking with your family/friends/colleagues? What aspects of the volunteer project did you talk about?

Have you had to talk about volunteering during job interviews or give any formal presentations on your experience? Do you ever discuss the ethics of projects you’ve been involved with in these contexts?

Do you feel there are aspects of your volunteer work that you find it hard to discuss with others?

### Appendix A.4. Conclusions

Is there anything you were expecting to talk about that we haven’t done?

Do you have anything you’d like to add to what we’ve already discussed?
